# Higher DNA repair in mitochondria of long-lived species

**DOI:** 10.18632/aging.203595

**Published:** 2021-09-27

**Authors:** Gustavo Barja

**Affiliations:** 1 Faculty of Biology, Complutense University of Madrid (UCM), Madrid, Spain

**Keywords:** longevity, aging rate, DNA repair, base excision repair, mitochondria

Positive correlation between genomic DNA repair and longevity has been classically described (reviewed in [1]). However, this corresponded to repair of exogenous DNA damage estimated as unscheduled DNA synthesis in fibroblasts (mitotic cells) after *in vitro* exposure to UV radiation. Such a correlation makes sense because a higher protection from exogenous damage, like that inflicted by UV radiation, is necessary in order for the already superior longevity potential of slowly aging animals to be phenotypically expressed. Long-lived animals can reach many decades of age only if the many different causes of early death due to extrinsic mortality (including UV-induced DNA damage and skin cancer) are avoided. That comparison can also be strongly biased by the much lower need of excision repair for removal of UV-induced lesions in rodents than in humans due to their fur and nocturnal habits.

Aging is an endogenous process that occurs in internal organs, most importantly in tissues containing postmitotic cells. Is the DNA repair of endogenous damage higher in long-lived animals? When base excision repair (BER) of genomic DNA was measured in four organs including heart and brain it was found not significantly changed or even decreased (instead of increased) in longer-lived caloric restricted mice [2]. Moreover, comparative studies in brain and liver of 15 mammalian and avian species have shown that repair of genomic DNA endogenous oxidative damage by BER in nuclear fractions does not correlate with longevity or, more frequently, is lower (instead of higher) in tissues of long-lived mammals when compared to short-lived ones [3]. BER plays an important role in repairing oxidative damage to DNA, but these results might indicate that genomic (almost all nuclear) BER does not play a key role in longevity extension. The negative correlation of genomic DNA BER with longevity is analogous to what was previously found for the endogenous total cellular antioxidant enzymes CuZn SOD, catalase, glutathione peroxidase, and reductase, as well as reduced glutathione, which most generally negatively correlate, and in some cases do not significantly correlate with longevity in mammals and vertebrates [4]. The likely evolutionary explanation for this is that the mitochondrial ROS production rate (mitROSp) is also lower in long-lived than in short-lived animals [4]. Since the mitochondria of long-lived animal species produce less H_2_O_2_ to the cytosol, they would also need less total cell endogenous antioxidants and less nuclear DNA repair systems. Endogenous total cell antioxidants and DNA repair enzymes are transitorily induced, when needed, to come back again to low levels when episodic increases in oxidative stress have been overcome. In this way, cells save much energy, which otherwise would be invested in the protein synthesis needed to continuously maintain high levels of cellular antioxidants and nuclear DNA repair enzymes when they are not needed at such high levels.

That is the situation concerning BER in nuclear DNA, but what occurs in the case of mitochondrial BER (mitBER)? MitBER had never been measured in species with different longevities, and we hypothesized that mitochondrial, instead of nuclear, BER is higher in long-lived than in short-lived mammals. We have thus recently measured activities and/or protein levels of various mitBER enzymes including DNA glycosylases, NTHL1 and NEIL2, and APE endonuclease in mitochondrial liver and heart fractions from eight mammalian species differing by 13-fold in longevity [5]. Our results show, for the first time, a positive correlation between mitBER and mammalian longevity. This suggests that the low steady-state oxidative damage in mitDNA of long-lived species, not observed for nuclear DNA, can be due to the combination of a low rate of damage generation (low mitROSp) and a high level of mitDNA repair (by mitBER) in these slowly aging animals. Further studies are needed to clarify if the same occurs for mitochondrial antioxidant enzymes. Indeed, the mitochondrial form of SOD, both MnSOD activity and protein level, is positively correlated with longevity in mammalian tissues [6]. Moreover, the only antioxidant overexpressor mouse that has shown a significant increase in maximum longevity was precisely the only one in which the antioxidant enzyme (catalase) was overexpressed inside the mitochondrial compartment, whereas mouse longevity did not increase when catalase was overexpressed in the peroxisome or in the nucleus [7]. Interestingly, indirect measurements in isolated mitochondria currently suggest that indeed mitochondrial antioxidants in general, in contrast to what happens for total cell ones [4], can also be present at higher levels in long-lived than in short-lived mammalian species [8]. If this is finally confirmed, three different factors working in the same direction would be responsible for the lower oxidative damage to mitDNA in the mitochondria of long-lived animals: (i) a low rate of mitROS generation; (ii) a high level of mitBER; and (iii) a high level of mitochondrial antioxidants? That situation would constitute a further case of cell compartmentation. The concentrations of sodium, potassium, calcium or protons strongly differ across cellular membranes by one or more orders of magnitude. Analogously, in long-lived animal species the BER enzyme levels are now known to be higher inside mitochondria than in the nucleus, and perhaps enzymatic and non-enzymatic antioxidants are present also at much higher levels inside mitochondria than at the cytosol. In any case, the recently described higher mitBER of long-lived mammals [5] seems to contribute to their superior longevity and constitutes a further piece of evidence indicating the special relevance of mitochondria and oxidative stress for the determination of species longevity.
